# Retention of Urine from Enlargement of the Third Lobe of the Prostate, Treated by Its Perforation

**Published:** 1831

**Authors:** 


### XXVIII.

### RETENTION OF URINE FROM ENLARGEMENT OF THE THIRD LOBE OF THE PROSTATE, TREATED BY ITS PERFORATION. By R. STAFFORD, Esq.

IN the analysis of Mr. Brodie's lectures on calculous diseases, published in the present number of this Journal, our readers will perceive that Mr. Brodie has perforated the third lobe of the prostate gland, for retention of urine occasioned by its enlargement. The eminent surgeon to whom we allude, recommends that, under certain circumstances, this operation should be performed. Mr. Stafford, whose little work on strictures has been formerly favourably noticed, has published two cases in which he performed this operation with success. As facts on such occasions are particularly valuable, we shall give an account of Mr. Stafford's cases.

*Case* 1. A gentleman, about 50 years of age, applied to Mr. Stafford with an impermeable stricture of the urethra, and slight enlargement of the prostate gland. It usually took him from two to four hours to empty the bladder, and he was subject to occasional attacks of retention of urine. Mr. Stafford gradually divided the stricture by means of the lancetted stilette, and in about *four months* the division was completed.

"After having permeated so great an extent of the urethra, the patient was attacked by retention of urine, which could not, however, be called *complete*, as he was able to void, though with great straining and extreme pain, about a teaspoonful of urine during the day. The bladder now became considerably distended; the urine was extremely fetid; and at the bottom of the vessel which contained it, there was deposited a muco-purulent sediment tinged with blood. Having completely cleared the urethra down to the prostate gland, I examined, *per anum*, the state of this organ, and found it to be rather more enlarged than on the first examination, and that on pressure with the finger it was extremely tender. The pulse at this time was hard and at 90; the tongue very dry and furred; the skin hot; and the countenance full of distress and anxiety.

He was ordered, (November 25 and 26,) to lose blood from the perineum by leeches and cupping, to make use of the warm bath, to foment, to take purgatives and opiates, and to use the latter in injections and suppositories. All these means failed to produce any effect, and the bladder was becoming so distended that there was reason to fear that it might burst, or urinary coma might come on, unless it was punctured; or unless the operation, which shall presently be described was performed. At this crisis, therefore, I requested to have another opinion, and my friend, Mr. Lawrence, surgeon to St. Bartholomew's Hospital, was called. The catheter could at this time be passed to the length of 8½ inches; and on examining its point, *per anum*, it appeared to have entered into the anterior part, or mouth of the prostate, and there to be obstructed. In this state of the case Mr. L. concurred with me in thinking it would be best to cut on through the obstruction, which appeared to be an enlargement of the third lobe of the prostate, with the lancetted stilette, in as near a line as possible in the natural channel into the bladder. Having introduced the instrument, I thrust forth the lancet at its point, and cautiously perforated the obstruction, until I advanced nearly two inches farther. The stilette now was eleven inches, but no urine flowed. It was therefore withdrawn, and a catheter was passed to the same point, but it would not go farther, until upon examination with the finger *per anum*, in order to discover its exact situation, it slipped into the bladder, and upwards of three pints of fetid urine were evacuated. In performing the operation the handle of the instrument was depressed as much as possible; and when the lancet which had made the cut had receded into its sheath, the blunt point was thrust in with the greatest caution until it had reached the extremity of the incision. The perforation was accomplished by protruding the lancet three times. The operation produced but little pain, and the bleeding was so trifling, that it could only be perceived by examining the point of the instrument. A No. 9 silver catheter was left in the bladder and secured, and the patient remained in bed. An opiate draught was administered. In the evening the pulse was reduced to 80—the skin had become moist, and the countenance had in a great measure lost the expression of anxiety."

We fancy that neither Mr. Stafford nor any body else will see many cases of retention so *complete*, that not a teaspoonful of urine is passed during the day. We cannot avoid remarking, en passant, that four months trial of the lancetted stilette, and retention of urine at the end of that time is no very great eulogium on the lancetted practice. For our own parts we believe that very few cases of stricture, very few indeed, but would yield in that time to the common means of dilatation, judiciously devised and dexterously executed. But this by the way.

We need not pursue the after details nor the after treatment. For a fortnight a gum catheter was kept in the bladder, and after this period it was withdrawn. In about three weeks after the operation he began to make water of his own accord, and in less than six he could completely empty the bladder. In January he returned into the country, and Mr. Stafford has since heard that he can void his urine as well as ever he did.

*Case* 2. A gentleman, aged 49, had laboured under symptoms of stricture for thirty years, with additional difficulty in passing his urine for five. The urine was alkaline, the bladder not completely evacuated. The natural orifice of the urethra was closed, but immediately beneath it was an opening connected with the canal. Half an inch anterior to the bulb was an impermeable stricture, and anterior to this a false passage which readily bled. The body of the prostate was slightly enlarged.

"After enlarging the orifice to its natural size, I gradually divided the diseased portion of the urethra, which extended about an inch and a half; and though in this case, as in the former, I proceeded with the greatest caution, for the reasons I have already mentioned, the operation was accomplished in four or five weeks. I found when I arrived at the prostate, that the instrument was still obstructed, and would not enter the bladder. The patient could make water in a larger stream; but he had still the same difficulty in expelling it, and the bladder never emptied itself. For these reasons, and as the catheter could be passed rather more than eight inches, and its point could be felt to have entered the prostatic portion of the urethra, I concluded this obstruction could be no other than an enlargement of the third or middle lobe; I therefore, as the symptoms were not very urgent, cautiously made three or four punctures in it. The patient felt some pain, and expressed himself as if something had been incised at the neck of the bladder. No bleeding ensued; and, indeed, so little did he suffer from it, that he felt no greater uneasiness than before the operation. In three days more I made the same number of punctures, which gave rise to exactly the same sensations at the time, but nothing more was felt. After the same interval of time, I again punctured the part, and on my fourth visit, my patient expressed himself considerably relieved. He could make water better, and he thought the bladder had gained more power. To ascertain the state of the urethra, I now introduced a steel sound, which passed easily into the bladder. On drawing off the urine afterwards, I discovered that the bladder could expel only half its contents. A catheter, therefore, was introduced night and morning, gradually increasing its size, until this organ completely regained its power. In about six weeks or two months the urinary organs of this patient were restored to their healthy function."

We think that, from the foregoing cases, as well as from those which are mentioned by Mr. Brodie, the puncture of an enlarged third lobe of the prostate, must be looked on under certain circumstances as a safe, if not an advisable operation. With respect to the lancetted stilette it would require much time, and many strong facts to enable us to pronounce a decided opinion on its advantages over the more ordinary means of dilatation for stricture. Of this we are certain, that Mr. Stafford employs it in many cases which might, and by other surgeons would, be successfully managed without it.

